# Early behavioral and physiological markers of social anxiety in infants with fragile X syndrome

**DOI:** 10.1186/s11689-021-09356-3

**Published:** 2021-03-20

**Authors:** Conner J. Black, Abigail L. Hogan, Kayla D. Smith, Jane E. Roberts

**Affiliations:** Department of Psychology, University of South Carolina, 1512 Pendleton Street, Barnwell College, Suite #220, Columbia, SC 29208 USA

**Keywords:** Fragile X syndrome, Social anxiety, Social behavioral inhibition, Biomarkers, Attention, Respiratory sinus arrhythmia, Physiology

## Abstract

**Background:**

Social anxiety is highly prevalent in neurotypical children and children with fragile X syndrome (FXS). FXS is a genetic syndrome that is characterized by intellectual disability and an increased risk for autism spectrum disorder. If social anxiety is left untreated, negative outcomes are highly prevalent later in life. However, early detection of social anxiety is challenging as symptoms are often subtle or absent very early in life. Given the prevalence and impairment associated with childhood social anxiety, efforts have accelerated to identify risk markers of anxiety. A cluster of early features of anxiety have been identified including elevated behavioral inhibition, attentional biases, and physiological dysregulation that index early emerging markers of social anxiety. Infants with FXS provide a unique opportunity to study the earlier predictors of social anxiety. The current study utilized a multi-method approach to investigate early markers of social anxiety in 12-month-old infants with FXS.

**Method:**

Participants included 32 infants with FXS and 41 low-risk controls, all approximately 12 months old. Parent-reported social behavioral inhibition was recorded from the Infant Behavior Questionnaire (IBQ-R). Direct observations of behavioral inhibition and attention were measured during a stranger approach task with respiratory sinus arrhythmia collected simultaneously.

**Results:**

Parent-reported social behavioral inhibition was not significantly different between groups. In contrast, direct observations suggested that infants with FXS displayed elevated behavioral inhibition, increased attention towards the stranger, and a blunted respiratory sinus arrhythmia response.

**Conclusions:**

Findings suggest that infants with FXS show both behavioral and physiological markers of social anxiety at 12 months old using a biobehavioral approach with multiple sources of input. Results highlight the importance of a multi-method approach to understanding the complex early emergent characteristics of anxiety in infants with FXS.

## Background

Anxiety is the most common childhood psychiatric disorder, with 8–20% of neurotypical (NT) children (i.e., children without a neurodevelopmental disability) diagnosed before adolescence [1–3]. If left untreated, childhood anxiety can lead to serious issues later in life such as depression, suicidality, substance abuse, and personality disorders [4]. However, low-cost intervention as early as preschool has been shown to produce long-term positive outcomes [5, 6] which fuels efforts to detect and treat early. Social anxiety is one of the most frequent and early emerging anxiety disorders, affecting nearly 10% of preschool-aged children [3]. Understanding the early predictors of social anxiety in infants and very young children can improve early identification, facilitate early intervention, and optimize long-term outcomes.

In NT infants and preschoolers, a number of prodromal indicators of social anxiety have been identified that develop in tandem with typical stranger fear. Behavioral inhibition, a temperament characteristic that includes features of fear, shyness, and withdrawal in response to novelty early in life, is one of the most robust predictors of later social anxiety symptoms and diagnoses [7–9]. For example, high behavioral inhibition across infancy, starting as young as 6 months of age, is a risk marker for social anxiety later in life [10]. Furthermore, preschool-aged children who demonstrate elevated and stable behavioral inhibition are up to four times more likely to be later diagnosed with social anxiety than children without elevated behavioral inhibition [7].

In addition to behavioral inhibition, several other factors have been identified that predict social anxiety, including atypical attention and physiological dysregulation. Atypical attention has been shown to contribute to the development and maintenance of anxiety symptoms [11]. However, this relationship is nuanced and varies by both type and severity of anxiety symptoms. For example, Waters and colleagues demonstrated that gaze towards threatening images (e.g., fearful or angry faces) only occurs with severe generalized anxiety symptoms [12, 13] whereas attention away from threatening images occurs in fear-based anxiety disorders such as social anxiety [14]. Thus, the relationship of attention to threat is complex and dependent on multiple factors.

In infancy, atypical physiological regulation has been linked with later negative outcomes including social anxiety. One measure of physiological regulation is respiratory sinus arrhythmia (RSA), which is a cardiac index of parasympathetic nervous system function that corresponds to respiration. RSA has been associated with multiple psychiatric disorders and maladaptive social behavior [15–17] as it plays an important role in physiological regulation of stress. Specifically, baseline RSA moderates the relationship between parental anxiety and an infant’s temperament with higher baseline RSA linked to the mother’s anxiety level whereas infants with low baseline RSA demonstrate no link between the mother’s anxiety and their temperament [18]. Moreover, infants with high RSA in a non-threatening environment demonstrated lower problem behavior compared to infants with high RSA in a disorganized environment who displayed a high level of problem behavior [19]. Likewise, greater suppression of RSA during a challenging event has been associated with reduced behavioral problems, improved emotion regulation, and decreased internalizing behaviors in preschoolers. Additionally, blunted RSA in response to a stressor is related to negative outcomes later in life [19]. In 6-month-old infants, atypical RSA regulation has been reported as a risk marker for increased likelihood of social anxiety during preschool [10]. These findings support an important role for elevated baseline RSA and RSA modulation as a protective factor for multiple child outcomes [18, 20].

Existing work demonstrates that prodromal signs of social anxiety are measurable early in life and that including multiple measures using a biobehavioral framework that integrates behavior with physiological regulation is a promising approach. Given the significant impairment associated with social anxiety, studies that refine our understanding of the early signs are critical to facilitate early detection and treatment. One productive way to approach this work is to study early risk factors in homogenous populations who are at high risk for social anxiety, such as fragile X syndrome (FXS), a genetic disorder with elevated rates of social anxiety [21, 22]. This study adopts a biobehavioral approach that integrates both biological and behavioral markers to advance our understanding of early signs and potential underlying mechanisms of social anxiety. We adopt elements from the neurovisceral integration theoretical model into our framework as it recognizes the role of autonomic regulation to cognitive, emotional, and behavioral function [21, 23]. Consistent with neurovisceral theory, the current study examined baseline and modulation of RSA, visual attention, and behavioral inhibition as potential factors associated with the expression of prodromal signs of social anxiety in a high-risk sample.

Fragile X syndrome is a monogenic disorder that affects approximately 1 in 3700 males and 1 in 6000 females [24]. It is caused by a CGG trinucleotide repeat expansion mutation of more than 200 repeats on the Fragile X Mental Retardation-1 (*FMR1*) gene on the X chromosome. Individuals with FXS often present with intellectual disability, maladaptive behaviors, and autism spectrum disorder [25, 26]. Notably, individuals with FXS are at increased risk for anxiety problems, with up to 86% of males meeting diagnostic criteria for at least one anxiety disorder [21]. Social anxiety is one of the most highly prevalent anxiety disorders in FXS, with 60.3% of males and 55.3% of females meeting diagnostic criteria for social anxiety [21, 22]. In addition to the high prevalence in both males and females, social anxiety is also cited as the most impairing phenotypic feature of children with FXS [27]. Clearly increased understanding of the onset and potential predictors of social anxiety in individuals with FXS is critical to identify and direct treatment to mitigate the long-term effects.

While research has clearly documented the high prevalence of social anxiety in FXS, little is understood about early signs of social anxiety in young children and infants with FXS. Research suggests that children with FXS display increased distress, gaze aversion, and avoidance during a social challenge task [27, 28]. Emerging evidence also suggests that elevated negative affect, a broader temperament profile that encompasses behavioral inhibition, in preschool-aged children with FXS is associated with anxiety symptoms later in development [29, 30]. Of note, this work has shown that elevated negative affect is selective in predicting anxiety symptoms and not features of autism spectrum disorder in FXS, which is important given that anxiety and autism spectrum disorder share features and are both commonly diagnosed in FXS [29, 30].

The role of attention and physiological regulation in relation to social anxiety in FXS has also not been well characterized. To date, there is evidence that preschoolers with FXS demonstrate reduced gaze towards novel people [27] and that school-age children demonstrate a greater attention bias towards angry over happy stimuli [31, 32]. Research has also documented reduced baseline RSA and blunted modulation of RSA in the context of cognitive [33] and social challenges [34] in FXS. Children and adolescents with FXS demonstrated dampened RSA suppression in response to emotionally evocative pictures [35], and preschoolers with FXS showed reduced suppression of RSA when completing a cognitively demanding task [33]. This work demonstrates reduced RSA modulation in individuals with FXS which is consistent with the “hyperarousal hypothesis” in FXS. The “hyperarousal hypothesis” postulates that a number of impairments and maladaptive outcomes in FXS are caused or exacerbated by elevated physiological arousal [36].

The current study used a biobehavioral approach to investigate social behavioral inhibition, attention, and physiological dysregulation in response to a stranger’s approach in 12-month-old infants with FXS. We include a comparison group of age-matched low-risk controls (LRC) to account for normal variation in stranger fear that is common in this developmental period. Multiple measures reflecting parent-reported behavioral inhibition, direct observations of behavioral inhibition, attention, and physiological regulation are used to provide a nuanced and comprehensive picture of prodromal social anxiety symptoms in infancy. Based on the high prevalence of anxiety in children and adults with FXS [21], we hypothesized that 12-month-old infants with FXS would exhibit prodromal markers of social anxiety manifested as elevated social behavioral inhibition, lower baseline RSA, and blunted RSA modulation in response to a novel person. Additionally, we hypothesized that indices of RSA would be related to behavioral responses based on research with infants who are neurotypical.

## Materials and methods

### Participants

Participants included a total of 73 infants who were 12 months of age. The primary group included 32 infants with FXS who were compared to 41 low-risk aged-matched neurotypical controls (see Table 1). FXS diagnosis was confirmed through genetic report provided by the parent revealing > 200 CGG repeats (i.e., full mutation FXS). LRCs were included based on absence of a family history of ASD or related disorders (e.g., tuberous sclerosis, FXS) and confirmation of typical development through study assessment, as detailed below. Exclusion criteria for both groups included gestation of < 37 weeks, presence of vision or hearing impairment, and lack of English proficiency within the home. Participants were recruited through a national registry for research and advertising through community resources as part of two larger longitudinal studies examining the emergence of autism spectrum disorder symptoms and anxiety in FXS (1R01MH107573-01A1, 2R01MH090194-06; PI: Roberts).

**Table 1:** Participant characteristics

	FXS (*n* = 32)	LRC (*n* = 41)	*p* value
*Key variables*			
Age, M (SD)	12.41 (1.49)	12.41 (.60)	.995
Number of females	10	9	.369
Mullen ELC, M (SD)	76.55 (21.52)	98.44 (12.83)	< .001
*Descriptive variables*^a^			
Race, *n*			
American Indian/Alaska Native	0	1	–
Asian	1	0	–
Black or African American	3	3	–
White	21	34	–
More than one race	8	2	–
Unknown	0	1	–
Ethnicity, *n*			
Hispanic or Latino	1	1	–
Not Hispanic or Latino	30	39	–
No answer	0	1	–
Household income, M (*n*)	62,715 (18)	66,657 (35)	–

^a^Descriptive variables were only used to characterize the sample. They were not used in any statistical analyses

### Measures

We utilized a multi-method, biobehavioral approach to characterize social behavioral inhibition and physiology. This included a parent report, two direct-observation measures (behavioral composite and visual attention) of social behavioral inhibition, and two measures of physiological regulation (RSA baseline and RSA stranger). Additionally, an RSA reactivity score was calculated to look at the modulation of RSA from baseline to stranger. We also included a measure of early cognition and development given that infants with FXS have documented developmental delays.

#### Parent report of social behavioral inhibition

The Infant Behavior Questionnaire-Revised (IBQ-R; 32 [37]) was used to assess parent-reported social behavioral inhibition. The IBQ-R is a 191-item temperament questionnaire that asks parents to rate how often their child responds to a variety of situations on a Likert scale from 1 to 7 (higher scores indicate higher frequency). The IBQ-R was normed on a neurotypical diverse sample of mother-child dyads and produced satisfactory internal consistency. The IBQ-R has been used widely across multiple samples of individuals with neurodevelopmental disabilities [30, 38]. While the IBQ-R has not been factor analyzed with a sample of infants with FXS, the factor structure of the Child Behavior Questionnaire (CBQ; which is the version of the temperament questionnaire designed for children between 3 and 7 years old, who have aged out of the IBQ) was investigated in FXS and found to follow a similar structure as the normative data [39]. In this study, we computed a social behavioral inhibition score that reflects fear in response to unfamiliar adults that has been done in previous work [10]. The score consisted of an average of eleven items and included questions such as “When introduced to an unfamiliar adult, how often did the baby cling to parent?”. In order to test for inter-item reliability, inter-item correlations were utilized and demonstrated strong consistency in responses, *r*s > .5.

#### Direct observation of social behavioral inhibition

The Stranger Approach paradigm from the Laboratory Temperament Assessment Battery (Lab-TAB) [40] was used to assess observed social behavioral inhibition and visual attention in response to the approach of an unfamiliar adult. The task consists of three parts: (1) approach of stranger towards the infant (10 s), (2) stranger kneeling in front of the infant with a neutral facial expression directed towards the infant (2 min), and (3) stranger withdrawing from the infant (10 s). The standard directions were followed [41] that involved informing the parent of the stages of the experiment with directions for the parent to remain neutral to allow the child to respond as “naturally” as possible. Infants were seated on their mother’s lap throughout the task and unrestrained. The stranger was a research assistant dressed in a black skirt, hooded sweatshirt, baseball cap, and sunglasses. In this paper, only the kneel portion of the paradigm was analyzed for two reasons. First, we believed that having the stranger in close proximity to the infant was likely the most stressful part of the experiment and we were interested in evoking the greatest fear response. Second, the kneel portion was the only epoch that had sufficient time to measure RSA given the parameters to edit and analyze the heart activity.

Videotaped recordings of the Stranger Kneel task were coded for escape behaviors, distress vocalizations, bodily fear, and visual attention using *Noldus The Observer XT* (version 10.0 software, Noldus Information Technology, Leesburg, VA, USA). Escape behavior, distress vocalizations, and bodily fear were coded for both intensity and duration. More details about the behavioral coding scheme can be found in Scherr et al. (2017). Composite scores were computed for escape behaviors, distress vocalizations, and bodily fear by multiplying the proportion of time spent at each intensity level with the intensity score, calculating a z-score for each behavior then averaging the z-scores together [42]. Visual attention was coded, and the proportion of time spent looking at the stranger was computed. Reliability was checked on 20% of videos, with Cohen’s kappa ranging from 0.81 to 0.90.

#### Physiological regulation

Heart activity was collected during a baseline period as well as during the stranger approach task. Electrocardiogram (ECG) data were recorded using a telemetry system that utilized two electrodes placed on the infant’s chest to collect the ECG signal at a sampling rate of 300Hz (Alive Technologies; CamNtech Ltd., Cambridge, UK). Trained research assistants visually inspected the ECG signal to identify and correct arrhythmias, false heart periods, and artifacts using CardioEdit software (Brain-Body Center, University of Illinois at Chicago). If > 10% of data were edited, the participant was excluded from further analyses. RSA was extracted via CardioBatch software (Brain-Body Center, University of Illinois at Chicago). CardioBatch samples sequential heart periods in 250-ms epochs and then utilizes a 21-point moving polynomial algorithm to de-trend the data. The data are filtered to remove variance associated with rate of respiration (.3–1.3 Hz), and RSA is then estimated by transforming the variance to its natural logarithm. Baseline RSA was computed as mean RSA during a 3-min baseline period during which the infant watched an engaging video. The engaging video included musical instruments and animal puppets with classical music playing with no people or language present. Baseline heart activity was collected before the start of the Lab-TAB. Stranger RSA was computed as mean RSA during the kneel phase of the stranger approach task. RSA reactivity was calculated by subtracting stranger RSA from baseline RSA.

#### Cognitive measure

The Mullen Scales of Early Learning [43] is a developmental measure used to assess cognitive abilities in children ages 0 to 68 months. The Mullen measures development across five domains: Gross Motor, Fine Motor, Receptive Language, Expressive Language, and Visual Reception. Four of the five sections, excluding Gross Motor, comprise an early learning composite (ELC) score with a mean of 100 and standard deviation of 15.

### Procedures

One-fourth of assessments took place in participants’ homes. There were no significant differences between those tested in their homes compared to those tested in the research laboratory on RSA reactivity, baseline RSA, age, household income, or behavior composites scores (*p* > .222). All assessments were completed by a team of two or three trained research specialists. Ethics approval was obtained for this study through the University of South Carolina Institutional Review Board, and parents signed an informed consent form prior to data collection. A brief developmental summary and monetary compensation was provided to the family upon completion of the assessment.

### Analytic plan

To assess differences between groups in parent-reported behavioral inhibition, observed behavioral inhibition, visual attention, baseline RSA, and Mullen ELC independent samples *t* tests were utilized (see Table 2). In order to examine differences in RSA across the kneel portion of the Stranger Task, within-group repeated-measures ANOVAs will be utilized. To determine within- and between-group differences in baseline RSA and stranger RSA, a repeated-measures mixed effects linear model was utilized via PROC MIXED in SAS 9.4. Condition (baseline, stranger) was specified as the repeated effect, nested within participant. Group, condition, Mullen ELC, sex, and the group by condition interaction were included as predictors, and a random intercept was included to account for individual differences in RSA. To describe the relationship between parent-reported behavioral inhibition, observed behavioral inhibition, and RSA variables, Pearson correlations were run in both groups.

**Table 2:** Means and standard deviations of key variables

Measure	FXS	LRC	*t* (df) = *t*, *p* = *p*, *d* = *d*
Parent-reported social behavioral inhibition	2.78 (1.06)	2.92 (1.01)	*t* (57) = 0.53, *p* = .600, *d* = .14
Social behavioral inhibition composite	− 0.19 (0.66)	0.062 (0.54)	*t* (63) = 1.82, *p* = .074, *d* = .45
Attentional allocation towards stranger	0.49 (.25)	0.40 (.13)	*t* (62) = − 1.95, *p* = .056, *d* = .47
Baseline RSA	4.21 (1.55)	4.53 (0.97)	*t* (41) = .778, *p* = .441, *d* = .23
Stranger RSA	4.17 (1.25)	3.86 (0.81)	*t* (41) = 1.42, *p* = .162, *d* = .40
RSA reactivity	0.04 (1.33)	0.68 (1.04)	*t* (41) = 1.77, *p* = .084, *d* = .54

Note: *t* = *t* value, *p* = *p* value, *d* = Cohen’s D

## Results

Analyses indicate that the FXS and LRC groups did not differ on the parent-reported social behavioral inhibition, (*t* (57) = .53, *p =* .600, *d =* .14). However, on the direct observation, the LRC group exhibited marginally greater social behavioral inhibition composite scores, (*t* (63) = 1.82, *p =* .074, *d =* .45) and more visual attention towards the stranger, (*t* (62) = − 1.95, *p =* .056, *d =* .47). Notably, these results reflect marginal *p* values but a medium effect size [44].

In order to assess differences within RSA values, multiple statistical analyses were completed. RSA from the kneel portion was examined for differences across the 2-min task. Results demonstrated that differences across the epochs were not observed in either the low-risk controls (*F* (2.16,58.17) = 2.14, *p* = .102) or infants with FXS (*F* (1.88,22.53) = 1.012, *p* = .375). As a result, the data was collapsed producing one RSA value for the entirety of the kneel portion. Additionally, to look at variability across groups, Levene’s test was completed. RSA variance across subject did not differ, F(1,41) = 1.28, *p* = .264.

**Fig. 1:**
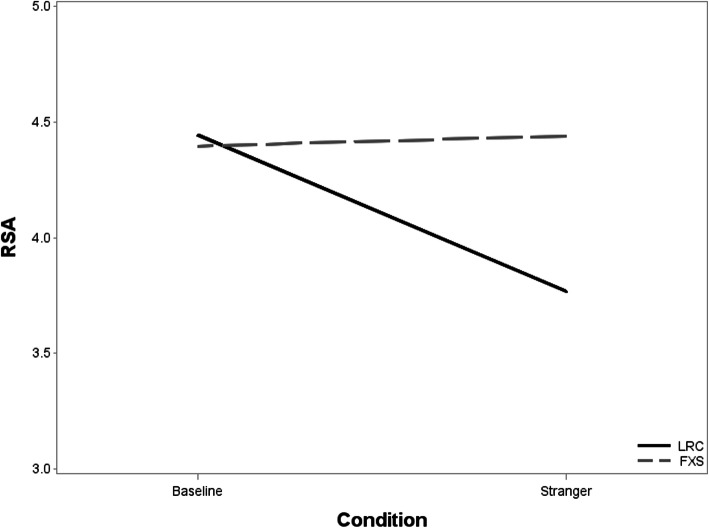
RSA by group across condition

Results from the repeated-measures mixed effects linear model indicated no significant main effects of group, *F* (1,40) = 1.04, *p* = .314, or condition (baseline and stranger), *F* (1,40) = 3.36, *p* = .074, but a significant group by condition interaction, *F* (1,40) = 4.18, *p* = .048. Due to the significant interaction, we examined within-group changes in RSA between baseline and stranger. The LRCs displayed a significant decrease from baseline to stranger, *t* (40) = − 3.15, *p* = .003 (mean change = 0.68) whereas the FXS group did not, *t* (40) = 0.13, *p* = .894 (mean change = 0.04; Fig. 1). Correlations between all dependent variables are displayed in Table 3. No significant correlations emerged between parent-reported social behavioral inhibition and the observed social behavioral inhibition composite. In the LRC group, a significant negative correlation emerged between RSA reactivity and proportion of time looking at the stranger.

**Table 3:** Correlations across key variables by group

FXS	1	2	3	4	5
1. Parent-reported social behavioral inhibition	1				
2. Social Behavioral Inhibition Composite	-.22	1			
3. Proportion of Time Looking at Stranger	-.08	-.35	1		
4. RSA Reactivity	.00	-.11	.31	1	
5. Mullen ELC	.19	-.07	.31	.41	1
LRC					
1. Parent-reported behavioral inhibition	1				
2. Behavioral Inhibition Composite	-.19	1			
3. Proportion of Time Looking at Stranger	.01	-.50**	1		
4. RSA Reactivity	.20	.31	-.42*	1	
5. Mullen ELC	-.20	-.07	.01	.01	1

* *p*<.05

** *p*<.01

## Discussion

Social anxiety is a highly prevalent and impairing condition. Understanding prodromal features of social anxiety in infancy can facilitate early intervention and mitigate negative long-term impacts. The present study is the first to examine social anxiety risk markers across multiple indices in infants with FXS, who are at elevated risk for comorbid social anxiety disorder. Evidence suggests that infants with FXS display both behavioral and physiological markers of social anxiety that are detectable as early as 12 months of age. However, these findings were nuanced and not consistent across all measures, highlighting the importance of a multi-method biobehavioral approach.

With regard to the direct observations, interesting group differences emerged that indicate distinct profiles. The LRC infant group exhibited an integrated biobehavioral response that is consistent with reports of existing work [10] implementing similar paradigms. This response was reflected through vocal distress, bodily movements, visual attention to social threat, and physiological modulation that was synchronized with their behavioral response (e.g., RSA suppression).

In contrast, the infants with FXS responded by subdued vocal and physical movement, increased visual attention to social threat, and a lack of physiological modulation that was not coupled with their behavioral response in comparison to LRC infants. Despite group differences in the modulation of RSA, the groups did not differ on baseline RSA. This was unexpected and could be related to age, as it has been reported that baseline differences do not emerge until later in life [45]. Our findings of elevated visual attention to the stranger in infants in this study, however, differ from a previous study with an independent older sample from our lab in which we reported reduced visual attention to the stranger in 4- and 5-year-old children with FXS [27]. The differences across studies could reflect some important developmental shifts in which increased attention to threat represents a less-mature response to a stranger during infancy that transitions to a reduced attention to threat over age. Evidence clearly highlights that it is trajectories of stranger fear from infancy across early childhood, rather than mean levels at any given age point, that are the most robust indicators of social anxiety [41] in NT children. Thus, future studies should address developmental stability or change in FXS from a longitudinal perspective.

These results indicate a complex picture, with varying patterns across different sources of information, highlighting the importance of a multi-method approach to identifying prodromal features of anxiety in infants. For example, results varied across sources of data with parent reports indicating that the groups were similar while direct observations demonstrated distinctions across groups. Likewise, the parent reports were not correlated with the direct observations or physiological markers whereas specific aspects of the direct observations were correlated with physiological markers in an informative and group-distinct manner. By including both parental and direct observations of behavior, we demonstrated the independence of these measures, and by including both direct observations and physiological measures, we report group distinctions in the synchrony between these measures that are informative.

Our findings have important implications. First, we report attention bias towards a social threat in the infants with FXS with nearly 50% of their time spent looking toward the stranger. Given that increased attentional vigilance is related to “distress” disorders, such as generalized anxiety disorder [12], our findings may suggest elevated risk for a more pervasive or generalized anxiety. However, since individuals with FXS are at an increased risk for social anxiety, generalized anxiety, and autism spectrum disorder, their looking pattern may change over time [27] or reflect a unique phenotypic feature that display increased risk for multiple types of anxiety and autism. Second, our finding that RSA suppression is reduced in the infants with FXS suggests that this may be a promising biomarker to detect risk for anxiety in this group. These findings are consistent with our previous work demonstrating that early physiological dysregulation could precede and/or contribute to atypical behavioral responses [46]. Moreover, our findings provide partial support of the hyperarousal hypothesis within FXS given their blunted RSA modulation. These findings could demonstrate the atypical physiological regulation may precede features of social anxiety or other clinical outcomes. These conclusions, however, are tentative given the inconsistent evidence across measures and moderate effect sizes. Additionally, the profile that emerges within infants with FXS may be unique since there is increased risk of multiple psychiatric disorders (i.e., autism spectrum disorder, social anxiety, GAD). Thus, these findings need to be empirically validated through larger samples, and developmental consequences of early prodromal differences should be investigated via longitudinal studies.

Strengths of the study include a biobehavioral approach that includes both behavioral and physiological markers along with multiple measures within each domain (e.g., two direct-observation measures). This approach is critical given the young age of our sample and the corresponding limited range of behaviors reflecting the developmental nature of social anxiety. In particular, the detection of early signs of anxiety are subtle and often absent in the first years of life with middle childhood as the typical age of diagnosis [47]. These issues are exacerbated by the complexity of the phenotype of FXS which includes developmental delays and social-communication deficits [48]. Finally, we included a well-matched LRC group to allow consideration of normative levels of social fear which are common at this age.

Despite these strengths, this study is limited by the use of only two groups, one of which is typically developing, which makes it impossible to conclude that profiles may be specific to FXS and not to neurodevelopmental disorder more broadly. Future studies should aim to explore these questions using a comparison group with developmental delay. Another limitation is that no outcome data was available for this study, and prodromal features were only measured at one timepoint. This is particularly important given evidence that high behavioral inhibition and atypical physiological responses across the first 3 years of life are robust predictors of anxiety outcome in NT children [10]. Future studies should investigate the complex relationship between mothers and children with FXS given both the increased prevalence of anxiety in mothers with the fragile X premutation who have children with FXS [49] and heritability of social anxiety from mother to child [50]. Thus, there could be a relationship between maternal anxiety and the presence or report of social anxiety symptoms in infants with FXS. Moreover, longitudinal studies should be conducted to examine the relationship of these early indicators of anxiety to outcomes including both anxiety and autism spectrum disorder given their overlapping features and frequent co-occurrence in FXS. Also, examining the relationship of these early markers to discrete anxiety disorders will be important as evidence suggests that elevated stranger fear in infancy may predict separation anxiety during early childhood which, in turn, is associated with social anxiety later in development [41].

## Conclusions

The present study is the first to use a biobehavioral approach to detect prodromal features of social anxiety in infants with FXS. Findings suggest that both behavioral and physiological signs are evident at 12 months of age in infants with FXS. These findings improve our understanding of the FXS phenotype in infancy as well as offer some of the first evidence for potential targets of intervention that have delivered promise in young typically developing children.

## Data Availability

The datasets analyzed during the current study are available from the corresponding author on reasonable request.

## Abbreviations

NT: Neurotypical
RSA: Respiratory sinus arrythmia
FXS: Fragile X syndrome
FMR1: Fragile X Mental Retardation-1
LRC: Low-risk control
IBQ-R: The Infant Behavior Questionnaire-Revised
CBQ: Child Behavior Questionnaire
Lab-TAB: Laboratory Temperament Assessment Battery
ECG: Electrocardiogram
ELC: Early learning composite
